# The Relationship of Aesthetic Dentistry Practices with Depression and Social Appearance Anxiety in Young Women

**DOI:** 10.3290/j.ohpd.b5714346

**Published:** 2024-08-27

**Authors:** Fikri Öcal, Yasemin Demirtaş Öcal

**Affiliations:** a DDS, PhD, Assistant Professor, Inonu University, Faculty of Dentistry, Department of Restorative Dentistry, Malatya, Turkey. The idea, hypothesis, experimental design, performed the experiments in partial fulfilment of requirements for a degree, wrote the manuscript.; b MA, Psychologist, Inonu University, Turgut Ozal Medical Centre. Performed and evaluated psychological tests, proofread the manuscript, and contributed substantially to the discussion.

**Keywords:** aesthetic treatment, depression, social appearance anxiety, women

## Abstract

**Purpose:**

This study aimed to evaluate the relationship between depression and social appearance anxiety in young female patients who applied for aesthetic dental treatment.

**Methodology:**

The study was conducted in a single centre and included 56 female patients aged 18–35 years. The mean age of the participants was 22.27 ± 5.62 years. Participants were informed before treatment, and the Beck Depression Inventory (BDI), Social Appearance Anxiety Scale (SAAS), and Visual Analogue Scale (VAS) were administered. Immediately after the treatment and 1 month later, the SAAS and VAS were administered again. The Kruskal–Wallis test was used for the comparison of the data obtained and the Friedman test was used for temporal comparison. Multiple comparisons were made with Dunn’s test.

**Results:**

After aesthetic dental treatment, all participants’ satisfaction with the appearance of their teeth increased, and social appearance anxiety decreased (p < 0.001). The group with the lowest pretreatment satisfaction level was the group with moderate/high-level depressive symptoms. There was no correlation between the severity of depression and the increase in satisfaction after treatment (r = 0.247; p = 0.066). Satisfaction levels were similar in all depression groups after treatment. The group with the highest level of social appearance anxiety before and after treatment was the group with moderate/high-severity depressive symptoms. A moderate positive correlation was found between depression severity and social appearance anxiety (r = 0.4; p = 0.002).

**Conclusions:**

The severity of depressive symptoms seen before treatment does not affect the level of satisfaction after treatment. There is a decrease in social appearance anxiety after aesthetic dental treatment. There is a positive relationship between social appearance anxiety and depression severity.

Physical appearance plays an important role in the formation of personality, and its relationship with self-perception begins early in life.^34^ Although the perception of attractiveness may be guided by innate preferences such as symmetry and ideal body proportions among women, the sociocultural environment also affects these preferences.^21^ One of the applications made to create an aesthetic appearance is the improvement of the face and teeth. The appearance of the teeth has an important place in the overall appearance of the face and plays an important role in social relationships.^25^ The desire for teeth to look white is among the main reasons for applications to dental clinics.^4^ The aesthetic appearance of teeth depends on their colour, shape, and position. In addition, other issues – such as the position of the lips and the appearance of the gums – also play an important role in an aesthetic smile.^29^ As a result, all components should be achieved together to achieve an aesthetic appearance.

Young women are applying to dental clinics with the demand for white and pearly teeth.^31^ Whitening treatment, direct resin composite application, and dental ceramics can be applied to make the teeth look whiter.^12^ Studies have shown that patients who consult dentists to improve the appearance of their teeth are motivated by the desire to increase attractiveness, regardless of structural and functional conditions.^31^ Although the concept of aesthetics is a difficult task to measure, the principles related to aesthetics and the perspective of aesthetics vary among societies.^35^ The good appearance of the teeth also means improved social relations, increased communication skills, and psychological well-being.^8^ In contrast, people with yellowed and decayed front teeth are not satisfied with their appearance. Studies show that people with these teeth are more dissatisfied with their lives.^13,39^ Aesthetic dental treatments have been said to improve patients’ quality of life and psychological state.^13^ It has also been stated that understanding the patient’s psychological state and expectations for dental treatment improves the physician–patient relationship and increases patient satisfaction.^1^

Dentists, past and present, have spent a significant amount of time treating the physical symptoms of patients with depression, anxiety, and other underlying psychological problems. Studies on patient satisfaction with treatment are generally conducted in orthodontic, orthognathic, and other surgical branches.^5,6,13^ Early and preventive treatment of health problems occurring in the oral and facial region is said to be very beneficial for the mental and mental health of individuals.^17,33^ People who apply to the clinic have opinions about the general appearance of their face and teeth, and these opinions affect how they will respond to treatment.^2,32^ In studies, personality traits, neuroticism and mental illnesses of the patients applying to the clinic affect the level of satisfaction after treatment.^15,30^ In the literature, there are studies examining patient satisfaction, such as patients’ expectations about the results of the treatment and how much they are interested in the treatment.^13,30,38^ In our study, we aimed to evaluate the relationship between the treatments applied to young female patients who applied for aesthetic dental treatment and depression and social appearance anxiety. At the end of the study, results regarding the relationship between the level of satisfaction after treatment, depression analysis, and social appearance anxiety will be obtained.

## MATERIALS AND METHODS

The study is a clinical research study and was conducted in a single centre June–December 2023.

### Ethical Approval

The study adhered to the Helsinki protocol and was conducted with the approval of the Inonu University Faculty of Medicine Malatya Clinical Research Ethics Committee (protocol number: 2023/46).

### Participants

Female participants aged 16–35 years, who could read and understand Turkish were included in the study. Inclusion criteria were vital and non-vital bleaching, diastema closure, composite veneers, and composite restoration of caries. Participants were asked to complete the Beck Depression Inventory (BDI), the Social Appearance Anxiety Scale (SAAS), and the Visual Analogue Scale (VAS), which expresses satisfaction with the appearance of their teeth.

### Tests

Participants completed the following tests.

#### Beck Depression Inventory (BDI)

The BDI developed by Beck et al is used to measure the level of depression and consists of 21 questions.^20^ It was adapted into Turkish by Hisli et al in 1988.^19^ The reliability coefficient (Cronbach Alpha) was reported as 80. Personal scale items are scored as 0 being the least and 4 being the most. The total score varies between 0–63. It can be applied to individuals over 15 years of age. 0–9 points are evaluated as minimal, 10–16 as mild, 17–29 as moderate, and 30–63 as level depressive symptoms.^20^

#### Social Appearance Anxiety Scale (SAAS)

The scale developed by Hart et al in 2008 is a five-point Likert-type scale consisting of 16 items.^18^ It was adapted into Turkish by Doğan in 2010.^14^ It was developed to measure the cognitive, emotional, and behavioural anxieties experienced in line with the evaluations to be made about the physical and social appearance of the person. Each question is given a score between 1 and 5. The total score that can be obtained is between 16 and 80. The higher the score obtained from the scale, the higher the social appearance anxiety.

#### Visual Analogue Scale (VAS)

This is a 100-mm line with a rating line ranging from 0 (unhappy) to 100 (very happy). These types of visual scales are widely used. They are used to measure health outcomes and are sensitive enough to record changes before and after an intervention.^40^

### Procedure

Patients were determined by the psychologist and dentist in the study group. Patients are invited to the study after they have been examined and decided on the need for aesthetic dental treatment. Participants have explained the details of the study at the face-to-face appointment and are provided with a verbal statement. They are assured that the treatment process and form will not be affected by their decision to participate and that the tests will remain anonymous and confidential. The patient ethics committee consent form and psychological tests (BDI, SAAS, and VAS) were provided after this stage and they were asked to fill them in the waiting room 10 min before the appointment. The anonymously completed tests were kept in a cabinet. After the completion of the treatment, participants completed the post-treatment questionnaires (SAAS-1 and VAS-1) in the waiting area. At the follow-up appointment 1 month later, SAAS-2 and VAS-2 were filled out again.

### Statistical Analysis

Data were analysed with IBM SPSS V23. Compliance with normal distribution was analysed by Shapiro–Wilk and Kolmogorov–Smirnov tests. Kruskal–Wallis test was used for the comparison of variables that did not conform to normal distribution according to groups, the Friedman test was used for the temporal comparison of variables that did not conform to the normal distribution in each group, and multiple comparisons were made with the Dunn test. The relationship between variables that did not fit the normal distribution was analysed by Spearman’s rho correlation coefficient. Analysis results were presented as mean ± standard deviation and median (minimum–maximum). The significance level was taken as p < 0.050.

## RESULTS

Sixty-five (65) participants were approached to participate in the study. All agreed to fill in the forms and receive treatment. Five (5) participants filled out the forms incompletely and 5 participants were not eligible for aesthetic dental treatment procedures. Finally, 56 participants were included in the study and evaluated. All participants were female, and the mean age was 22.27 ± 5.62 years. See Table 1 for the aesthetic dental treatments performed on the participants.

**Table 1:** The mean age of the participants and aesthetic dental treatments applied

Gender	Women	Number	Per cent (%)
		56	100
Applied aesthetic dental treatments	Diastema closure Composite veneer Caries restoration Bleaching	29 14 9 4	51.8 25 16.07 7.14

The mean BDI score of the participants was 10.88 (see Table 2). According to the results of the BDI, 58.9% of the participants had minimum-level depressive symptoms, 19.6% had mild, 16.1% had moderate, and 5.4% had level depressive symptoms.

**Table 2:** Participants’ mean BDI scores and categorisation according to depression status

	Mean ± sd	Median (min–max)
Beck Depression Inventory Mean Score	10.88 ± 8.4	9 (0–37)
	Number	Per cent (%)
Depression status		
Minimum-level depressive symptoms	33	58.9
Mild-level depressive symptoms	11	19.6
Moderate/High-level depressive symptoms	12	21.5

A statistically significant difference was found between the median values of VAS scores measured at different times (p < 0.001). The median VAS score was 60, the median VAS-1 score was 90 and the median VAS-2 score was 90 (Table 3). Here, the difference was observed between the VAS score before treatment and the scores at other times.

**Table 3:** Comparison of VAS and SAAS scores of all study participants before treatment, immediately after treatment, and 1 month after treatment

	Mean ± sd	Median (min–max)	Test statistics	p*
VAS	59.91 ± 27.54	60 (–100)b	73.322	<0.001
VAS-1	86.79 ± 15.68	90 (40–100)^a^		
VAS-2	87.23 ± 17.63	90 (0–100)^a^		
SAAS	29.54 ± 11.87	26.5 (16–69)^b^	16.667	<0.001
SAAS-1	27.95 ± 12.09	24 (16–78)^a,b^		
SAAS-2	26.88 ± 10.7	24.5 (16–61)^a^		

*Friedman test; a–b: There is no difference between times with the same letter.

A statistically significant difference was found between the median values of the SAAS score measured at different times (p < 0.001). The median SAAS score was 26.5, the median SAAS-1 score was 24, and the median SAAS-2 score was 24.5. Here, there was a difference between the SAAS pretreatment score and the 1-month post-treatment score (Table 4).

**Table 4:** Comparison of age, BDI, VAS, and SAAS scores of participants grouped according to depression level

	Groups			Test statistics	p*
	Minimum-level depressive symptoms	Mild-level depressive symptoms	Moderate/High-level depressive symptoms		
Age			22.25 ± 4.85	1.143	0.565
		20 (16–35)	21 (16–35)		
BDI	5.58 ± 2.91	11.91 ± 2.02	24.5 ± 6.07	43.023	<0.001
	6 (0–9)^b^	12 (10–15)a	22 (18–37)^a^		
VAS	64.85 ± 25.23	60.91 ± 26.72	45.42 ± 31.44	3.281	0.194
	70 (0–100)^B^	65 (10–100)B	50 (0–100)^B^		
VAS-1	89.55 ± 11.75	82.27 ± 18.89	83.33 ± 21.14	1.176	0.555
	90 (60–100)^A^	90 (40–100)A.B	92.5 (50–100)^A^		
VAS-2	89.7 ± 14.14	85.91 ± 9.7	81.67 ± 28.87	2.351	0.309
	90 (40–100)^A^	90 (70–100)A	90 (0–100)^A^		
Test statistics	47.784	9.000	17.892		
p**	<0.001	0.011	<0.001		
SAAS	26.21 ± 9.86	30.82 ± 7.24	37.5 ± 16.43	7.149	0.028
	24 (16–54)	32 (19–42)B	33 (16–69)		
SAAS-1	24.76 ± 9.81	28.91 ± 8.56	35.83 ± 16.87	7.879	0.019
	20 (16–50)^b^	33 (16–43)a.b. A.B	34 (19–78)^b^		
SAAS-2	24.85 ± 9.14	25.36 ± 7.13	33.83 ± 14.7	4.100	0.129
	23 (16–55)	22 (17–39)A	32.5 (16–61)		
Test statistics	6.019	14.308	1.733		
p**	0.049	0.001	0.420		

* Kruskal–Wallis test; ** Friedman test; Mean ± standard deviation; Median (minimum–maximum); a–b: There is no difference between groups with the same letter; A–B: There is no difference between times with the same letter in each group.

When comparisons between groups were analysed;

A statistically significant difference was found between the median values of the BDI score according to the groups (p < 0.001). While the median score in the group with minimum-level depressive symptoms was 6, the median score in the group with mild-level depressive symptoms was 12, and the median score in the group with moderate/high-level depressive symptoms was 22. Here, the score in the group with minimum-level depressive symptoms differed from the other groups.

A statistically significant difference was found between the median values of the SAAS score according to the groups (p = 0.028). The median score in the group with minimum-level depressive symptoms was 24, the median score in the group with mild-level depressive symptoms was 32, and the median score in the group with moderate/high-level depressive symptoms was 33. No difference was observed here as a result of pairwise comparisons.

A statistically significant difference was found between the median values of the SAAS-1 score according to the groups (p = 0.019). The median score was 20 in the group with minimum-level depressive symptoms, 33 in the group with mild-level depressive symptoms, and 34 in the group with moderate/high-level depressive symptoms. Here, the scores in the group with minimum-level depressive symptoms differed from the scores in the group with moderate/high-level depressive symptoms.

Other variables did not show a statistically significant difference between the groups.

When VAS, VAS-1 and VAS-2 scores were analysed in each group.

In the group with minimum-level depressive symptoms, a statistically significant difference was found between the VAS score median values (p < 0.001). The median VAS score was 70, the median VAS-1 score was 90, and the median VAS-2 score was 90. The difference was observed between the VAS score before treatment and the scores at other times.

In the group with mild to level depressive symptoms, a statistically significant difference was found between the VAS score median values (p = 0.011). The median VAS pretreatment score was 65, the median VAS-1 score was 90 and the median VAS-2 score was 90. The difference was observed between VAS and VAS-2 scores.

A statistically significant difference was found between the VAS score median values in the group with moderate/high-level depressive symptoms (p < 0.001). The median VAS pretreatment score was 50, the median VAS-1 score was 92.5, and the median VAS-2 score was 90. The difference was observed between the VAS score before treatment and the scores at other times.

In the group with minimum-level depressive symptoms, a statistically significant difference was found between the median values of the SAAS score (p = 0.049). The median SAAS score was 24, the median SAAS-1 score was 20, and the median SAAS-2 score was 23. No difference was found here as a result of pairwise comparisons.

In the group with mild-level depressive symptoms, a statistically significant difference was found between the median values of SSI score (p = 0.001). The median SAAS score was 32, the median SAAS-1 score was 33 and the median SAAS-2 score was 22. The difference was observed in the pretreatment and post-treatment first month values.

There was no statistically significant difference between the VAS-1/VAS score difference median values according to the groups (p = 0.112). The median score of the group with minimum-level depressive symptoms was 20, the median score of the group with mild-level depressive symptoms was 20, and the median score of the group with moderate/high-level depressive symptoms was 37.5. There was no statistically significant difference between the VAS-2/VAS score difference median values according to the groups (p = 0.293). The median score of the group with minimal-level depressive symptoms was 20, the median score of the group with mild-level depressive symptoms was 20, and the median score of the group with moderate/high-level depressive symptoms was 35. There was no statistically significant difference between the VAS-2/VAS-1 score difference median values according to the groups (p = 0.851). The median score of the group with minimum-level depressive symptoms was 0, the median score of the group with mild-level depressive symptoms was 0, and the median score of the group with moderate/high-level depressive symptoms was 0. There was no statistically significant difference between the median values of SAAS-1/SAAS score difference according to the groups (p = 0.817). The median score of the group with minimum-level depressive symptoms was –1, the median score of the group with mild-level depressive symptoms was –2, and the median score of the group with moderate/high-level depressive symptoms was –1. There was no statistically significant difference between the median values of SAAS-2/SAAS score difference according to the groups (p = 0.078). The median score of the group with minimum-level depressive symptoms was –1, the median score of the group with mild-level depressive symptoms was –4, and the median score of the group with moderate/high-level depression was –3.5. There was no statistically significant difference between the median values of the SAAS-2/SAAS-1 score difference according to the groups (p = 0.106). The median score of the group with minimum-level depressive symptoms was 0, the median score of the group with mild-level depressive symptoms was –2, and the median score of the group with moderate/high-level depressive symptoms was –2.5 (Table 5).

**Table 5:** Comparison of depression groups according to time periods before, after, and 1 month after treatment

	Groups			Test statistics	p*
	Minimum-level depressive symptoms	Mild-level depressive symptoms	Moderate/High-level depressive symptoms		
VAS-1/VAS	24.7 ± 22.32	21.36 ± 23.67	37.92 ± 25.89	4.38	0.112
	20 (0–100)	20 (–10–80)	37.5 (0–100)		
VAS-2/VAS	24.85 ± 23.9	25 ± 28.81	36.25 ± 27.23	2.452	0.293
	20 (0–100)	20 (–20–90)	35 (0–100)		
VAS-2/VAS-1	0.15 ± 8.52	3.64 ± 15.83	–1.67 ± 16.56	0.323	0.851
	0 (–20–30)	0 (–20–30)	0 (–50–20)		
SAAS-1/SAAS	–1.45 ± 5.71	–1.91 ± 2.77	–1.67 ± 9.5	0.403	0.817
	–1 (–22–10)	–2 (–6–3)	–1 (–18–15)		
SAAS-2/SAAS	–1.36 ± 5.66	–5.45 ± 4.61	–3.67 ± 15.72	5.106	0.078
	–1 (-12 - 9)	–4 (–17–0)	–3.5 (–21–38)		
SAAS-2/SAAS-1	0.09 ± 6.26	–3.55 ± 4.8	–2 ± 11.05	4.49	0.106
	0 (-20–14)	–2 (–17–1)	–2.5 (–26–23)		

*Kruskal–Wallis test; Mean ± standard deviation; Median (minimum–maximum).

There is a statistically significant positive moderate relationship between the BDI and SAAS pretreatment scores (r = 0.4; p = 0.002). There is a statistically significant positive moderate relationship between the BDI and SAAS-1 scores (r = 0.421; p = 0.001). There is a statistically significant positive weak correlation between BDI and SAAS-2 scores (r = 0,354; p = 0,007). There was no statistically significant relationship between the BDI score and other variables (p > 0.050) (Table 6).

**Table 6:** Examination of the relationship between BDI and other variables

	BDI	
	r	p
Age	–0.007	0.960
VAS	–0.247	0.066
VAS-1	–0.120	0.380
VAS-2	–0.149	0.274
SAAS	0.400	0.002
SAAS-1	0.421	0.001
SAAS-2	0.354	0.007

r: Spearman’s rho correlation coefficient.

## DISCUSSION

All of the participants were female, and the average age represented a young population. All of them came to the clinic to have their teeth look aesthetic and white. Women are more interested in their physical appearance than men and have more aesthetic treatments to change their appearance.^31^ When this situation is analysed according to age distribution, younger people receive more aesthetic treatments than older people.^3^ Women are more critical about the appearance of teeth, which have an important place in facial aesthetics, and they apply to dental clinics more for aesthetic appearance.^24,36^ In this context, more than half of the aesthetic dental treatment applications to our clinic were for diastema closure. People with diastemas teeth may be perceived as less attractive, less intelligent and from a lower social class in society.^27^ Reis et al and Ren et al stated that closing the gaps between the teeth is a treatment that significantly changes the smile.^27,28^ As a result, having an aesthetic smile can create a more attractive feeling. Satisfaction levels increased and social appearance anxiety decreased after cosmetic dental treatment in all participants in the study. The level of satisfaction with the teeth decreased in people who showed symptoms of high-severity depression. Regarding this situation, Paans et al reported that there was a positive correlation between depression level and dissatisfaction with the body.^26^ It has been stated that aesthetically bad-looking teeth and teeth with malocclusion increase social and psychological anxiety.^10^ Mental disorders such as depression and anxiety may show changes immediately or 1 month after treatment.^7,9^

In our study, the level of satisfaction increased over time in all depression groups. This suggests that satisfaction with dental treatment may be high regardless of the severity of depression. In the literature, there are few studies evaluating the relationship between satisfaction with aesthetic dental treatments and mental illnesses. The degree to which individuals are satisfied with their teeth at the beginning of treatment and the mental states they experience may be determinants of satisfaction after treatment. Sarin et al evaluated the effects of personality traits on treatment outcomes and reported that neurotic personality type negatively affected post-treatment satisfaction.^30^ Neuroticism is a personality trait and is an important finding in terms of affecting treatment outcomes. As a result, a significant relationship could not be established between the level of depression experienced and the level of satisfaction with the treatment.

Social appearance anxiety is defined as the anxiety that an individual feels when his/her general appearance is evaluated by others.^23^ Orofacial appearance is an important part of an individual’s image and is closely related to self-confidence.^17^ There was a positive correlation between the severity of depression experienced by the participants in the study and social appearance anxiety. In the evaluation, social appearance anxiety increased as the severity of depression increased. Similarly, Çelik et al reported a positive correlation between depression and social appearance anxiety, similar to the findings in our study.^11^ When the time-dependent change after aesthetic dental treatment was analysed, social appearance anxiety decreased in all depression groups. The aesthetic appearance of the teeth of the individuals may have caused a decrease in social appearance anxiety. Regarding this situation, Kalyoncuoğlu et al reported that missing teeth or poor appearance of teeth increased social appearance anxiety.^22^ Social appearance anxiety may be closely related to depression, anxiety, and personality traits.^11^ They said that the anxiety in individuals decreased after the aesthetic dental treatment and this situation contributed to the decrease in depression.^16,37^

In our age, the desire to look beautiful with the influence of social media and society causes a common aesthetic search in young women. Our study focused on examining the relationship between young women’s satisfaction with their teeth and the depression and social appearance anxiety they experience. The satisfaction of individuals after aesthetic dental treatments affects the patient’s life as well as the decision-making process of clinicians. Individuals’ satisfaction after treatment may be closely related to their personality structure and mental illnesses. In the results obtained from the study, depression severity, and social appearance anxiety are two closely related disorders. It can significantly affect the satisfaction of individuals with the appearance of their teeth. Aesthetic dental treatments may be useful in reducing social appearance anxiety. People in the group with moderate/high-level depressive symptoms may become as satisfied with their teeth as others. In this respect, the depression experienced by the individuals and their satisfaction levels after treatment are similar. 

Our study has some limitations. One of them is that the number of participants is 56 and it is only about women. More reliable results can be obtained by increasing the sample size and including men. Another limitation is whether the severity of depression that existed before the study will change at the end of the study. In follow-ups longer than 1 month, the extent to which depression status and social appearance anxiety change can be examined. The strengths of our study are the evaluation of satisfaction levels after aesthetic dental treatment applications together with mental illnesses. Another strength is that the satisfaction levels and social appearance anxiety scores immediately after the treatment were also evaluated 1 month after the study and an evaluation could be made according to the mental changes.

## CONCLUSIONS

The results obtained from this study show that after aesthetic dental treatment, regardless of the severity of depression, the level of satisfaction with the appearance of the teeth increases and social appearance anxiety decreases. Another result is that people with high depression symptoms experience higher social appearance anxiety. In the measurements made 1 month after the treatment, the level of satisfaction and social appearance anxiety is similar to the situation immediately after the treatment, regardless of the depression status. The severity of depression does not affect the satisfaction with aesthetic dental treatment.
